# A Nationwide Evaluation of the Prevalence of Human Papillomavirus in Brazil (POP-Brazil Study): Protocol for Data Quality Assurance and Control

**DOI:** 10.2196/31365

**Published:** 2022-01-05

**Authors:** Jaqueline Driemeyer Correia Horvath, Marina Bessel, Natália Luiza Kops, Flávia Moreno Alves Souza, Gerson Mendes Pereira, Eliana Marcia Wendland

**Affiliations:** 1 Escritório de Projetos Programa de Apoio ao Desenvolvimento Institucional do Sistema Único de Saúde Hospital Moinhos de Vento Porto Alegre Brazil; 2 Department of Chronic Conditions Diseases and Sexually Transmitted Infections Health Surveillance Secretariat Ministry of Health Brasília Brazil; 3 Department of Community Health Universidade Federal de Ciências da Saúde de Porto Alegre Porto Alegre Brazil

**Keywords:** quality control, quality assurance, evidence-based medicine, quality data

## Abstract

**Background:**

The credibility of a study and its internal and external validity depend crucially on the quality of the data produced. An in-depth knowledge of quality control processes is essential as large and integrative epidemiological studies are increasingly prioritized.

**Objective:**

This study aimed to describe the stages of quality control in the POP-Brazil study and to present an analysis of the quality indicators.

**Methods:**

Quality assurance and control were initiated with the planning of this nationwide, multicentric study and continued through the development of the project. All quality control protocol strategies, such as training, protocol implementation, audits, and inspection, were discussed one by one. We highlight the importance of conducting a pilot study that provides the researcher the opportunity to refine or modify the research methodology and validating the results through double data entry, test-retest, and analysis of nonresponse rates.

**Results:**

This cross-sectional, nationwide, multicentric study recruited 8628 sexually active young adults (16-25 years old) in 119 public health units between September 2016 and November 2017. The Human Research Ethics Committee of the Moinhos de Vento Hospital approved this project.

**Conclusions:**

Quality control processes are a continuum, not restricted to a single event, and are fundamental to the success of data integrity and the minimization of bias in epidemiological studies. The quality control steps described can be used as a guide to implement evidence-based, valid, reliable, and useful procedures in most observational studies to ensure data integrity.

**International Registered Report Identifier (IRRID):**

RR1-10.2196/31365

## Introduction

Data quality assurance is essential to maintain the credibility and internal validity of a study and to enable further generalization of the results [1]. Quality control must be the basis of any work process for guaranteeing process standardization, resource maximization, and loss reduction and costs [2].

Research protocols traditionally include some tools to control sampling and measurement errors during their execution [3]. High-quality data and effective data quality assessments are required to measure the real impact of interventions and outcomes. The process of quality control of epidemiological studies is usually briefly described or not described in detail despite being an important step to ensure the reliability of the results. Although many studies have used quality assurance and quality control procedures, few have described those procedures in enough detail to support other researchers’ ability to improve research quality [4-8]. Therefore, detailed descriptions of the quality control process should be more widely discussed among researchers.

It is recommended that protocols follow at least 3 steps: planning and standardization, planned implementation, and process analysis [3]. Although all of these steps are critical, one of the most important factors is based on the planning and standardization of procedures [9-11]. Standardized procedures are reflected in bias reduction [9] and reliable data [12]. Data standardization is essential when large and integrative studies are increasingly prioritized [13]. In addition, standardized surveys are able to provide comparable data across populations or periods [14].

Thus, this paper aims to describe the stages of quality control of the POP-Brazil study [15] and to present an analysis of the quality indicators. The POP-Brazil study was designed to provide representative data on human papillomavirus (HPV) prevalence in young adults who use the public health system in all 26 Brazilian state capitals plus the Federal District of Brasilia. High-quality public health data are needed to facilitate decision making and planning at all levels of the health system, monitor program performance, justify financial support, and, especially, provide data to evaluate the HPV vaccine program in a continental country, such as Brazil. To ensure standardization in all centers and the quality of the data produced by the POP-Brazil study, a quality control plan was carried out, with control points and key quality indicators [16].

## Methods

### Overview

The POP-Brazil study is a cross-sectional, nationwide, multicentric study [15]. Large-scale population studies face difficulties in recruiting representative samples [17]; thus, the initial planning involved a series of agreements with all 26 Brazilian state capitals plus the Federal District of Brasilia to choose the primary care health centers (ie, those with appropriate infrastructure and serving a diverse local population).

**Figure 1:**
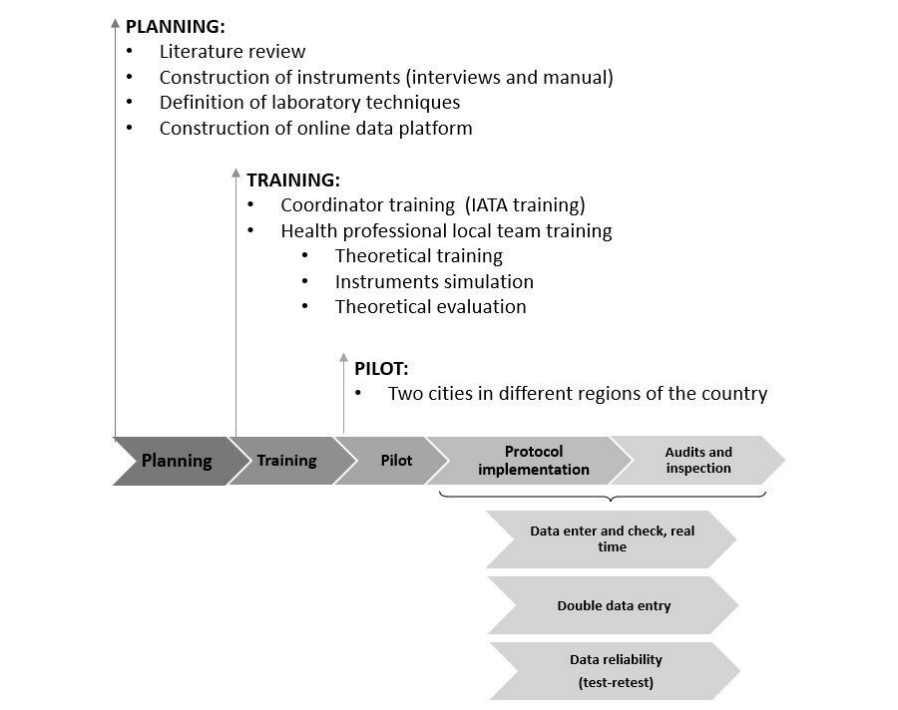
Quality control protocol strategies of the POP-Brazil study. IATA: International Air Transport Association.

All quality control protocol strategies are presented in Figure 1, and these points will be explored and discussed one by one.

### Planning

The planning phase included a comprehensive literature review [18] to obtain support for the development of the research protocol, construction of data collection instruments (structured interviews), and definition of laboratory techniques to be used. These actions are part of quality assurance, meaning they are activities planned and systematically implemented to provide confidence that the study will meet quality requirements.

The structured interview guide was developed through adaptations of previously validated questionnaires, followed by consultation with sexual behavior experts and pretests [19,20]. The questions accessed in the interview can be found in Multimedia Appendix 1. A part of the questionnaire was validated as the first instrument able to describe the knowledge, beliefs, and behaviors regarding HPV and related subjects [20].

In addition, an operation manual was constructed, consisting of specific instructions for conducting the interview, collecting biological samples, storing and transporting samples, and entering data on the online data platform (Sisepidemio—built exclusively for the project). Additionally, the manual contained guidance for the correct photographic recording of suspected oral or genital HPV lesions.

### Training

Municipal health departments from each city invited a health professional to be the local study coordinator. Local study coordinators were responsible for packing and shipping the biological material, as well as organizing the study logistics at the local level. To ensure the safe transportation of biological samples and their quality [21-23], all coordinators were trained and certified by the International Air Transport Association since reliability and generalization of search results depend on data collection methods [24].

All 250 health professionals involved in data collection were trained, in loco, by the research group. This training was divided into theoretical and practical phases. Theoretical training, lasting 4 hours, presented the procedures to be performed and simulated the collection of biological material with anatomical models. For practical training, when the centers were visited by the researchers from the central team, the professionals responsible for data collection participated in an interview simulation (recorded for analysis) [9].

During the visit to the centers, their structural suitability for biological sample collection and storage was analyzed, and the logistics for transportation of these samples were defined. In all centers, the samples were kept at a temperature below 25 °C inside a refrigerator or portable coolers with reusable artificial ice. To ensure that the collected biological samples were maintained at adequate temperature, a thermometer close to the samples automatically measured the temperature every 30 seconds. All professionals received a temperature control worksheet, where the thermometer temperature should be noted daily, for future audits.

A theoretical evaluation was performed at the end of training and served as the basis to verify the adequacy of the training. This evaluation was composed of 10 questions regarding training focal points. All professionals scored at least 82.7%. A minimum score of 70% was considered as a quality indicator.

At the end of the data collection, the professionals performed this evaluation again and scored at least 83.4% (*P*=.01). The second evaluation was used to verify the retention of knowledge and the maintenance of procedure adequacy throughout the data collection process. These evaluations helped to ensure standardization in all centers, since standardization must be treated as a priority for guaranteed quality [25].

When the differences between each question were analyzed, no significant differences were found among them, with the exception of one question regarding the handling of biological specimens, where an increase in the knowledge of the collectors was observed during the research period (51.0% to 72.5%; *P*=.02). For these analyses, a Cohen kappa coefficient was calculated using SAS software (SAS Institute Inc, Cary, NC), version 9.4.

### Pilot Study

The pilot stage is crucial for the adequacy of any research protocol. The pilot can provide recommendations to avoid or minimize observed errors and optimize logistics and quality management [9]. The adequacy of the methodology for collecting data and biological material, the functioning of the online platform, and the logistics and security of the sample shipment were verified.

It was identified that the penile material collection methodology needed to be improved. The initial choice of technique was not retaining sufficient biological material for penile HPV detection. First, it was proposed that the collection be performed by health professionals through friction of the epithelium using a Digene “brush” (Qiagen, Hilden, Germany) moistened with saline. This technique was changed to self-collection under the guidance and supervision of a health care professional, with a moistened Dacron swab (Qiagen). Apparently, self-collected specimens produced a better [26] or equal [27] proportion of sufficient specimens than physician-collected specimens for penile samples. The data entry platform was enhanced to accommodate changes in the biological material logistics process.

### Protocol Implementation

To standardize procedures and harmonize work conduct with the aim of increasing management efficiency and meeting the required demands, logistics systems for material transport, as well as for the inventory and systematic storage of samples, were created. All materials sent to public health units were cataloged and monitored by tracking codes. Participant data were collected on paper and typed on an online platform. The biological materials were sent to Porto Alegre through a logistics company by air weekly. All barcode-identified control worksheets, materials, and questionnaires were returned to the technical team in Porto Alegre.

The control of samples was performed through an online platform using barcode identification. Upon arrival at the laboratory in Porto Alegre, samples were recorded and visually inspected for volume, tube integrity, presence of blood, and particles, and any inadequacy was recorded in the online platform. The oral samples were stored without processing at –80 °C in 2 aliquots for future DNA extraction and analysis. The genital samples were centrifuged and aliquoted in 2 cryovials for storage at –80 °C. DNA extraction and analyses were gradually performed as previously described [15].

### Audits and Inspection

All public health units received at least one monitoring visit (an audit) for quality control of the study. The audit aimed to identify any inadequacies in the collection, storage, or registration of data in the online platform. In case of delays in data collection or when inadequacies were found, new training was performed, and new centers or new professionals were included.

During the audit visits, researchers from the technical team supervised at least one data collection (interview and biological material) from a participant. All visits were documented through photographic records and report production.

Temperature controls were checked by auditing the thermometer graphics along with the temperature control worksheets. Each thermometer generated a temperature variation graph that was analyzed to ensure proper storage of biological materials. The database was also monitored, with a daily backup of the online platform. As a quality indicator, the temperature should always be below 25 °C.

In the POP-Brazil study, the nonresponse rates (when the respondent reported that they preferred not to answer or report that they did not know or remember) were monitored and are presented in Table 1. The only significant difference found between genders was regarding contraception. A response rate of 95% was considered good quality, based on a previous study [28], assuming that nonrespondents have similar characteristics as respondents.

**Table 1:** Nonresponse rates by question groups in the POP-Brazil study.

Variable	Total, n (%)	Women, n (%)	Men, n (%)	*P* value
Sociodemographic	1096 (15.29)	830 (15.32)	266 (5.26)	.97
Sexual behavior (symptoms and practices)	761 (8.17)	544 (3.75)	217 (4.43)	.12
Sexual health	656 (7.28)	501 (3.77)	155 (3.50)	.77
Women’s sexual health	287 (3.23)	—^a^	—	—
Drugs	185 (2.82)	131 (2.36)	54 (3.30)	.19
Contraception	93 (1.26)	35 (0.25)	58 (2.30)	<.001

^a^This variable applied to women only, so no comparison was conducted.

### Double Data Entry

The reliability of a procedure can be defined as the ability to achieve the same results (with minimal variations) when the same procedure is performed by a different person or at a different time. To test the reliability of data entry, we opted for double-checking data validation: All questionnaires were typed on an online platform by health professionals and digitized using optical brand recognition by the technical team. In cases of disagreement in any answer, corrections were made using the answers written on the paper questionnaire as the gold standard.

We used Remark Office OMR 2014 v.9.5 software (Gravic Inc, Malvern, PA) for optical brand recognition. Questionnaires were scanned using ScanSnap Manager v.6.5 (Fujitsu Global, Tokyo, Japan) and processed in Remark. Additionally, manual validation was performed during the scan. Manual validation occurred when Remark did not recognize some answer in the scanned questionnaire. Under these circumstances, Remark highlights the variable for manual validation.

The error rate (inconsistencies) was calculated based on the total number of inadequacies by the total number of answers. Additionally, we analyzed survey responses to ensure nonduplication [29]. The first comparison between Sisepidemio and Remark was performed when 10% of the total sample was reached, to test the effectiveness of these systems. An error rate of 2.67% was observed. A second comparison between the databases was performed to verify the overall quality of the data produced by POP-Brazil. From 2100 questionnaires, an average inconsistency of 0.71% (range 0% to 4.37%) was found. Date of birth was the variable with the highest typing error rate.

### Test-Retest

Additionally, data reliability was analyzed by comparing a first application of the interview, conducted by a health professional, to a second application of the interview, conducted by the technical team via telephone. The average time between test-retest was 166.17 (SD 69.5) days and ranged from 1 month to 14 months.

The calls were standardized. For this, a manual for telephone interviews was used. This manual outlined each step of the call (conducting the interview and how to present the questions), as well as highlighting confidentiality issues. The telephone interview was conducted to confirm the validity of previously obtained data and included part of the main questionnaire (29 of 65 questions). The questions chosen for this second interview were those with answers that are easy to remember or do not change over time (eg, date of birth). Calls were made on alternate weekdays and shifts, with at least 3 calls to each participant before classifying them as a “noncontact.”

From the total sample, 20% of patients were randomized to be enrolled in this quality control step. The reliability of the test and retest questions was measured using kappa coefficients [30,31]. To classify the degree of concordance, the criteria by Landis and Koch [31] were used: excellent: >0.74; good: 0.59 to 0.74; moderate: 0.40 to 0.58; and poor: <0.40. A minimum sample size of 173 interviews was estimated based on the Cohen kappa coefficient value [32] of the variable “race” (κ = 0.63), with a power of 80% and a 2-tailed alpha of .05. Overall, the agreement between the test and retest was considered good (kappa range across questions = 0.59 to 0.74).

The rate of inconsistencies was also calculated, as previously mentioned. A total of 1311 individuals were contacted, and 448 interviews were completed. The effective percentage of contact with the participant through phone calls was, on average, 34.17% (448/1311), with most calls being classified as “noncontact” (843/1311, 64.30%). When we obtained contact for the interview, the confirmation rate of participation in the POP-Brazil study was high: 95.7% (448/468) confirmed participation and agreed to answer the survey again. Only 14 individuals confirmed participation in the survey but refused to answer again (14/468, 3.0%); 6 individuals reported not remembering being part of the POP-Brazil survey.

## Results

The POP-Brazil study protocol was approved by the Moinhos de Vento Hospital research board (Approval Number 1607032) and was designed in accordance with the 1964 Helsinki declaration and its later amendments. The pilot study was conducted in 2 cities in different regions of the country (north: Rio Branco; south: Curitiba) between September 2016 and December 2016. A total of 8628 sexually active young adults (16-25 years old) were enrolled in 119 public health units between September 2016 and November 2017 [15].

## Discussion

Inaccurate reporting of data hampers the generalizability and correct interpretation of results from scientific papers, so assessing research quality and susceptibility to bias is essential when interpreting and conducting studies. Large multicenter epidemiological studies are the cornerstone of evidence-based medicine, so their design, logistics, and quality processes should always be disclosed to ensure data integrity. A broad discussion of quality processes among epidemiological studies, such as the POP-Brazil study, is important to ensure data reliability and to highlight the necessity of the process in observational studies.

Epidemiological data, as in other types of research, are susceptible to variations that can lead to inappropriate conclusions. For this reason, quality control is critical in conducting any study, and the integrity of the study results is determined by the quality of the collected data [33]. Although the importance of quality assurance and control in epidemiological studies is consistent among researchers and widely discussed, few studies have been written about the results of quality control [33,34] or described the tools applied. Moreover, although it is expected that studies, especially larger ones, perform quality control, there is no information about the process in smaller or observational studies.

In the present study, several steps were mentioned. Among these factors, we highlight the importance of conducting a pilot study that provides the researcher the opportunity to refine or modify the research methodology and to develop large-scale studies [35]. A well-conducted pilot will encourage methodological rigor and ensure that the work is scientifically valid and publishable [36,37]. Although pilots play an important role in any research, the reality is that they receive little or no attention in many studies. Few articles, epidemiology, or research textbooks cover the topic with the necessary detail [35]. The pilot study provided important information regarding penile HPV sample collection, allowing us to intervene appropriately to correct the process and ensure the quality of the collected samples.

Another fundamental factor is validating the results through double data entry, test-retest, and analysis of nonresponse rates. Double data entry is considered the criterion standard for minimizing data entry errors [38], and the rate was low in this study (2.67%). Automated form processing is a valid alternative to double manual data entry [39]. It is noteworthy that, in general, there are no differences in the use of computerized systems and manual systems regarding the quality of the final data obtained [38,39]. However, the efficiency of brand recognition systems has recently been evidenced, thereby providing more cost-effective and operationally efficient systems [38,39]. Test-retest findings should not be used as a single quality control measure since contact attempts were mostly unsuccessful (64.30%). Lack of contact is a common finding in this type of data collection. Herath et al [40] reported that 78% of respondents did not respond to phone calls. Another study using a similar methodology reported a noncontact rate of approximately 60% [41,42]. The refusal to participate in the interview (2.99%) was similar to that presented by a large Brazilian epidemiology study (3.8%) [43]. This same survey observed that approximately 22.8 calls were needed to obtain a full interview [43]. Our effectiveness rate was higher, requiring 8.54 calls for a full interview. Although the test-retest estimate was considered generally good and similar to previous studies [44], some questions showed lower agreement, such as reporting sexually transmitted infections and drug use. The retest emphasized the relevant time frame (for example, “Did you smoke at the time you answered the POP-Brazil survey?” or “Did you start to smoke, after the survey?”), but it is possible that the participant would not remember the answer he or she had previously given due to the time gap between the tests. It is recognized that longer recall periods result in less accurately reported estimates [45,46]. However, there is no definition of the appropriate length of the recall period, and it also depends on the type of information, individual characteristics such as cognitive ability or socioeconomic variables, and the nature of the survey [45].

The nonresponse level is considered a central indicator of data quality, but little is known about the possible bias caused by nonresponse. Few studies check this parameter, and nonresponse rates vary between surveys [28,47], which may lead to bias in estimates [24,48,49] because it is dependent on the sample population [24]. Important differences between survey responders and those who do not answer some questions may lead to a bias associated with nonresponse that impacts the generalizability and validity of the study findings. In POP-Brazil, most of the nonresponses were about not knowing or remembering some answers, and these variables depended on participant memory rather than a refusal to answer such questions.

In conclusion, quality control processes are a continuum, not restricted to a single event, and are fundamental to the success of data integrity and to minimizing bias in epidemiological studies. A number of useful items has been discussed in this report. The quality control steps described can be used as a guide to implement evidence-based, valid, reliable, and useful procedures in most observational studies to ensure data integrity.

## Appendix

Multimedia Appendix 1 Topics accessed in the interview.

## Abbreviations

HPV: human papillomavirus

## References

[1] Anglemyer A, Horvath H, Bero L (2014). Healthcare outcomes assessed with observational study designs compared with those assessed in randomized trials. Cochrane Database Syst Rev.

[2] Ministério da Saúde (2006). Assistência farmacêutica na atenção básica: instruções técnicas para sua organização.

[3] Groves RM, Heeringa SG (2006). Responsive design for household surveys: tools for actively controlling survey errors and costs. J Royal Statistical Soc A.

[4] Chen H, Hailey D, Wang N, Yu P (2014). A review of data quality assessment methods for public health information systems. Int J Environ Res Public Health.

[5] Parsa N, Zibaeenezhad MJ, Trevisan M, Karimi Akhormeh A, Sayadi M (2020). Magnitude of the quality assurance, quality control, and testing in the Shiraz Cohort Heart Study. Biomed Res Int.

[6] Xavier M, Baptista H, Mendes JM, Magalhães P, Caldas-de-Almeida JM (2013). Implementing the World Mental Health Survey Initiative in Portugal - rationale, design and fieldwork procedures. Int J Ment Health Syst.

[7] Boing AC, Peres KG, Boing AF, Hallal PC, Silva NN, Peres MA (2014). EpiFloripa Health Survey: the methodological and operational aspects behind the scenes. Rev Bras Epidemiol.

[8] Kessler RC, Berglund P, Chiu WT, Demler O, Heeringa S, Hiripi E, Jin R, Pennell B, Walters EE, Zaslavsky A, Zheng H (2004). The US National Comorbidity Survey Replication (NCS-R): design and field procedures. Int J Methods Psychiatr Res.

[9] Hansen SE, Benson G, Bowers A, Pennell BE, Lin YC, Duffey B, Hu M, Hibben KC Survey Quality. Guidelines for Best Practice in Cross-Cultural Surveys.

[10] Biemer PB, Lyberg LE (2003). Introduction to Survey Quality.

[11] Tolonen H, Koponen P, Mindell J, Männistö S, Kuulasmaa K (2014). European Health Examination Survey-towards a sustainable monitoring system. Eur J Public Health.

[12] Dewitt J, Capistrant B, Kohli N, Rosser BRS, Mitteldorf D, Merengwa E, West W (2018). Addressing participant validity in a small internet health survey (The Restore Study): protocol and recommendations for survey response validation. JMIR Res Protoc.

[13] McLaughlin PM, Sunderland KM, Beaton D, Binns MA, Kwan D, Levine B, Orange JB, Peltsch AJ, Roberts AC, Strother SC, Troyer AK (2021). The quality assurance and quality control protocol for neuropsychological data collection and curation in the Ontario Neurodegenerative Disease Research Initiative (ONDRI) study. Assessment.

[14] Mindell JS, Giampaoli S, Goesswald A, Kamtsiuris P, Mann C, Männistö S, Morgan K, Shelton NJ, Verschuren WMM, Tolonen H, HES Response Rate Group (2015). Sample selection, recruitment and participation rates in health examination surveys in Europe-experience from seven national surveys. BMC Med Res Methodol.

[15] Wendland E, Caierão J, Domingues C, Maranhão AGK, Moreno Alves de Souza F, Hammes L, Falavigna M, Hilgert J, Hugo F, Bessel M, Villa L, Benzaken A (2018). POP-Brazil study protocol: a nationwide cross-sectional evaluation of the prevalence and genotype distribution of human papillomavirus (HPV) in Brazil. BMJ Open.

[16] Juran J, Godfrey AB (1999). Juran's quality handbook, 5th edition.

[17] Downes M, Gurrin L, English D, Pirkis J, Currier D, Spittal M, Carlin JB (2018). Multilevel regression and poststratification: a modeling approach to estimating population quantities from highly selected survey samples. Am J Epidemiol.

[18] Colpani V, Bidinotto AB, Falavigna M, Giozza SP, Benzaken AS, Pimenta C, Maranhão AGK, Domingues CMAS, Hammes LS, Wendland EM (2016). Prevalence of papillomavirus in Brazil: a systematic review protocol. BMJ Open.

[19] Saulle R, Miccoli S, Unim B, Semyonov L, Giraldi G, de Vito E, Ficarra MG, Firenze A, Gregorio P, Boccia A, La Torre G (2013). Validation of a questionnaire for young women to assess knowledge, attitudes and behaviors towards cervical screening and vaccination against HPV: survey among an Italian sample. Epidemiology Biostatistics and Public Health.

[20] Horvath JD, Kops NL, Caierão J, Bessel M, Hohenberger G, Wendland EM (2018). Human papillomavirus knowledge, beliefs, and behaviors: A questionnaire adaptation. Eur J Obstet Gynecol Reprod Biol.

[21] Pereira MEC (2006). Transporte externo de material biológico. Fundação Oswaldo Cruz. Curso de Gestão da Qualidade, Biossegurança e Ambiente (QBA-on line).

[22] Aires CAM, Araujo CFMD, Nobre ML, Rusak LA, Assis UGD, Lopéz DCM, Franco VDC, Heringer M, Silva APD, Portilho MM, Pereira MEDC, Soeiro MDNC (2015). Biossegurança em transporte de material biológico no âmbito nacional: um guia breve. Rev Pan-Amaz Saude.

[23] (2017). ANEXO II - Resolução CFM no 2169/2017. Conselho Federal de Medicina.

[24] Van Loon A (2003). Survey non-response in the Netherlands effects on prevalence estimates and associations. Annals of Epidemiology.

[25] Saarnak CF, Utzinger J, Kristensen TK (2013). Collection, verification, sharing and dissemination of data: the CONTRAST experience. Acta Trop.

[26] Hernandez BY, McDuffie K, Goodman MT, Wilkens LR, Thompson P, Zhu X, Wong W, Ning L (2006). Comparison of physician- and self-collected genital specimens for detection of human papillomavirus in men. J Clin Microbiol.

[27] Ogilvie GS, Taylor DL, Achen M, Cook D, Krajden M (2009). Self-collection of genital human papillomavirus specimens in heterosexual men. Sex Transm Infect.

[28] Mindell J, Tipping S, Pickering K, Hope S, Roth M, Erens B (2010). The effect of survey method on survey participation: analysis of data from the Health Survey for England 2006 and the Boost Survey for London. BMC Med Res Methodol.

[29] Grey JA, Konstan J, Iantaffi A, Wilkerson JM, Galos D, Rosser BRS (2015). An updated protocol to detect invalid entries in an online survey of men who have sex with men (MSM): how do valid and invalid submissions compare?. AIDS Behav.

[30] Webber M, Huxley P, Harris T (2011). Social capital and the course of depression: six-month prospective cohort study. J Affect Disord.

[31] Landis JR, Koch GG (1977). The measurement of observer agreement for categorical data. Biometrics.

[32] Gao Y (2012). Using SAS to Determine the Sample Size on the Cohen’s Positive Kappa Coefficient Problem. MWSUG 2012 Conference Proceedings.

[33] Harel O, Schisterman E, Vexler A, Ruopp M (2008). Monitoring quality control: can we get better data?. Epidemiology.

[34] Kim SM, Choi Y, Choi BY, Kim M, Kim SI, Choi JY, Kim S, Song JY, Kim YJ, Kee M, Yoo M, Lee J, Park BY (2020). Prospective cohort data quality assurance and quality control strategy and method: Korea HIV/AIDS Cohort Study. Epidemiol Health.

[35] Thabane L, Ma J, Chu R, Cheng J, Ismaila A, Rios LP, Robson R, Thabane M, Giangregorio L, Goldsmith CH (2010). A tutorial on pilot studies: the what, why and how. BMC Med Res Methodol.

[36] Doody O, Doody CM (2015). Conducting a pilot study: case study of a novice researcher. Br J Nurs.

[37] Lancaster G, Dodd S, Williamson P (2004). Design and analysis of pilot studies: recommendations for good practice. J Eval Clin Pract.

[38] Fifolt M, Blackburn J, Rhodes D, Gillespie S, Bennett A, Wolff P, Rucks A (2017). Man versus machine: comparing double data entry and optical mark recognition for processing CAHPS survey data. Qual Manag Health Care.

[39] Paulsen A, Overgaard S, Lauritsen JM (2012). Quality of data entry using single entry, double entry and automated forms processing--an example based on a study of patient-reported outcomes. PLoS One.

[40] Herath H, Weerasinghe N, Weerarathna T, Hemantha A, Amarathunga A (2017). Potential use of telephone-based survey for non-communicable disease surveillance in Sri Lanka. BMC Public Health.

[41] Kertscher B, Speyer R, Fong E, Georgiou AM, Smith M (2015). Prevalence of oropharyngeal dysphagia in the Netherlands: a telephone survey. Dysphagia.

[42] Kontto J, Tolonen H, Salonen AH (2020). What are we missing? The profile of non-respondents in the Finnish Gambling 2015 survey. Scand J Public Health.

[43] Vigilância das Doenças e Agravos Não Transmissíveis e Promoção da Saúde. Ministério da Saúde.

[44] Griep S, Santos SM, Cardoso LDO, Fonseca MDJMD, Alves MGDM, Souto EP, Chor D (2013). Capital social no ELSA-Brasil: confiabilidade teste-reteste do Resource Generator scale. Rev. Saúde Pública.

[45] Kjellsson G, Clarke P, Gerdtham U (2014). Forgetting to remember or remembering to forget: a study of the recall period length in health care survey questions. J Health Econ.

[46] Stull DE, Leidy NK, Parasuraman B, Chassany O (2009). Optimal recall periods for patient-reported outcomes: challenges and potential solutions. Curr Med Res Opin.

[47] Lynn P, Clarke P (2002). Separating refusal bias and non-contact bias: evidence from UK national surveys. J R Statist Soc D.

[48] Berlin NL, Hamill JB, Qi J, Kim HM, Pusic AL, Wilkins EG (2018). Nonresponse bias in survey research: lessons from a prospective study of breast reconstruction. J Surg Res.

[49] Christensen A, Ekholm O, Glümer C, Juel K (2014). Effect of survey mode on response patterns: comparison of face-to-face and self-administered modes in health surveys. Eur J Public Health.

