# Supplementary material for: A Nationwide Evaluation of the Prevalence of Human Papillomavirus in Brazil (POP-Brazil Study): Protocol for Data Quality Assurance and Control
Source: JMIR Res Protoc. 2022 Jan 5;11(1):e31365. doi: 10.2196/31365 (PMC8771346; doi:10.2196/31365)
Supplement: Multimedia Appendix 1 [file resprot_v11i1e31365_app1.docx]

**Supplement file 1.**

Topics accessed in the interview.

| Topics | Specific Content |
| --- | --- |
| Identification of participant | Name; Mother's name; Birth date; Identification document; Biological sex; Zip code; Telephone number; “How many times have you attended the public health unit in the last 12 months?” |
| Sociodemographic variables | Race/skin colour; Relationship status; Education level; Last-month family income and the number of people dependent on this income; Employment status; Socioeconomic class. |
| Smoking, alcohol and other drugs | Current and past smoking habits, including quantity and frequency; Use of alcohol and illicit drugs (types and frequency); Alcohol and drug use before and/or during sexual intercourse. |
| Reproductive health | Age of menarche; Use of contraceptive methods; Pap testing history and if so, whether any alterations have ever been observed. |
| Sexual behaviour | Sexual orientation; Gender identity; Age at first sexual intercourse; Age of the partner who had first sexual intercourse; Gender(s) of sexual partners; The frequency of condom uses and the number of sexual partners (at last 5 years and 2 months). |
| Health | History of contraception, pregnancy, and childbirth; History of STIs; HIV testing history; STI knowledge and beliefs; Suspicious HPV-related lesions (oral and/or genital). |
| HPV and vaccination knowledge | If the participant received the HPV vaccine; Reason for not getting vaccinated; How much participants know about HPV and vaccination (when and who must receive the vaccine); Source of information about HPV vaccination. |
